# Microleakage of conventional light-cure resin-based fissure sealant and resin-modified glass ionomer sealant after application of a fluoride varnish on demineralized enamel

**DOI:** 10.1371/journal.pone.0208856

**Published:** 2018-12-11

**Authors:** Concepción Germán-Cecilia, Sandra María Gallego Reyes, Amparo Pérez Silva, Clara Serna Muñoz, Antonio José Ortiz-Ruiz

**Affiliations:** Department of Integral Paediatric Dentistry, Faculty of Medicine and Dentistry, University of Murcia, Murcia, Spain; University Lyon 1 Faculty of Dental Medicine, FRANCE

## Abstract

**Background:**

International guidelines on the prevention of caries recommend sealing of the pits and fissures of the permanent molars. There is no evidence on which type of material is most effective on demineralized enamel.

**Aim:**

To evaluate the microleakage of a conventional light-cured, resin-based fissure sealant (LCRBS), GrandiO Seal, and a resin-modified glass ionomer sealant (RMGIS), Vitremer, after application of a fluoride varnish, Bifluorid 12, on demineralized enamel.

**Design:**

80 human third molars were divided into eight groups. The groups combined the three study factors (1) type of enamel (intact or demineralized); (2) enamel non-varnished or varnished with Biflourid12; and (3) type of sealant (GrandiO Seal or Vitremer). The percentage of microleakage after thermocycling was measured using imaging analysis software. The Kruskal-Wallis plus Dunn tests were used to compare differences in microleakage in the different groups.

**Results:**

The lowest microleakage was in the unvarnished groups, and was the same for GrandiO Seal and Vitremer. When varnish was applied, microleakage was greater in demineralized enamel than in intact enamel for both LCRBS and RMGIS.

**Conclusion:**

The application of fluoride varnish on demineralized enamel increases the microleakage of both GrandiO Seal and Vitremer.

## Introduction

The Global Burden of Disease (GBD) 2015 study [1] found that untreated caries in the permanent teeth was the most highly-prevalent condition measured, (age-standardized prevalence: 34.1%), affecting 2.5 billion people worldwide (95% UI: 2.4 to 2.7 billion). In the US, the prevalence of dental caries in first and permanent teeth in the 2–19 years age group was 45.8% [2].

The greatest risk of caries lesions in permanent teeth occurs during the first years after eruption, due to the lack of posteruptive maturation of the enamel [3] and they most frequently appear on the pits and fissures of the first molars, where they appear even before eruption is complete, because the anatomy favours the formation and retention of biofilm [4].

International dentistry and paediatric dentistry guidelines recommend sealing the primary and permanent molars in children and adolescents to prevent the onset of cavities and minimize the progression of noncavitated occlusal carious lesions [5,6].

Currently, two main types of pit and fissure sealants are used, those based on resins and those based on glass ionomer. LCRBS are mainly based on bisphenol A glycidyl methacrylate (BIS-GMA) [7]. Sealants based on glass ionomer cements, which contain and release fluorine, include presentations in the form of conventional low and high viscosity glass ionomers, and RMGIS [8].

The latest Cochrane review [9] on the use of pit and fissure sealants for the prevention of caries in the permanent dentition concluded that the use of LCRBS in permanent molars reduces the risk of caries at 48 months compared with unsealed molars (70% vs. 18.9%), and there are no consistent studies that establish the effectiveness of using glass ionomer sealants in the prevention of occlusal caries in molars versus unsealed molars. Neither is there scientific evidence on which type of sealant (resin-based or glass ionomer) is more effective in preventing caries [10].

Sealant retention seems to be the determining factor in the expected prevention of caries. LCRBS have a retention rate of 80% at 24 months, 70% at 54 months and 39% at 9 years [11]. Low viscosity ionomers only retained 4% at 48 months and RMGIS and high viscosity ionomers, particularly those used with atraumatic restorative treatment, have a higher retention rate [12]

The association between the risk of caries and complete loss of retention of pit and fissure sealants is significant with LCRBS, but not with glass ionomer sealants, probably due to their ability to release fluorine [13]. Frencken and Wolke [14] showed that, although detachment of the ionomer was observed clinically, the sealing material was retained at the bottom of the pits and fissures microscopically, with the sealing material exerting its preventive effect at the bottom of the cavity.

Microleakage is one of the biggest drawbacks of sealants, as it causes bacterial invasion and secondary caries [15,16]. Salivary contamination during the placement of LCRBS increases microleakage and reduces retention. Due to the greater tolerance of ionomers to moisture, glass ionomer sealants would be a good alternative in situations where good insulation is difficult during the application of the sealant, such as when there is complicated management or partial eruption of a permanent molar [17].

RMGISs have a higher tolerance to humidity and incorporate resin particles that increase resistance to wear and fracture. They have a twin adhesion mechanism, consisting of small cement tags and a hybrid layer, micromechanical interlocking, mainly in dentin, and true chemical bonding with the formation of ionic bonds between the functional carboxylate groups of the ionomer and the calcium ions on the surface of the hydroxyapatite. Adhesion is improved by the use of etching acid and an adhesive or primer [18].

The topical application of fluoride varnishes 2–4 times a year seems to be effective in preventing caries in the temporary and permanent dentition [19,20]. However, the review by Ahovuo-Saloranta et al [21] found low quality evidence of the benefits of LCRBS and fluoride varnish over fluoride varnish alone and of the benefits of glass ionomer sealants versus fluoride varnish.

The objective of this study was to assess whether the application of a fluoride varnish influenced microleakage of an LCRBS or RMGIS placed on demineralized enamel.

## Materials and methods

### Teeth

The study protocol was approved by the Research Ethics Committee of the University of Murcia (Spain) (ID: 2148/2018). All patients were adults and were informed of the study and gave written informed consent to participate. Eighty third molars in perfect condition extracted by the Oral and Maxillofacial Surgery Service of the General University Hospital Reina Sofía (Murcia, Spain) for orthodontic reasons or due to pericoronitis were used. Once extracted, the teeth were stripped of calcified deposits by ultrasound, and bone and periodontal remains by curettes. Once cleaned, they were immersed in a thymol solution 0.1% for 24 hours and then kept in distilled water which was changed daily until use, which was never more than 30 days after extraction. The teeth were varnished with two layers of nail polish (Resist and Shine, L’Oreal, Paris, France) leaving the pits and fissures of the occlusal face free.

### Experimental groups

**Fig 1:**
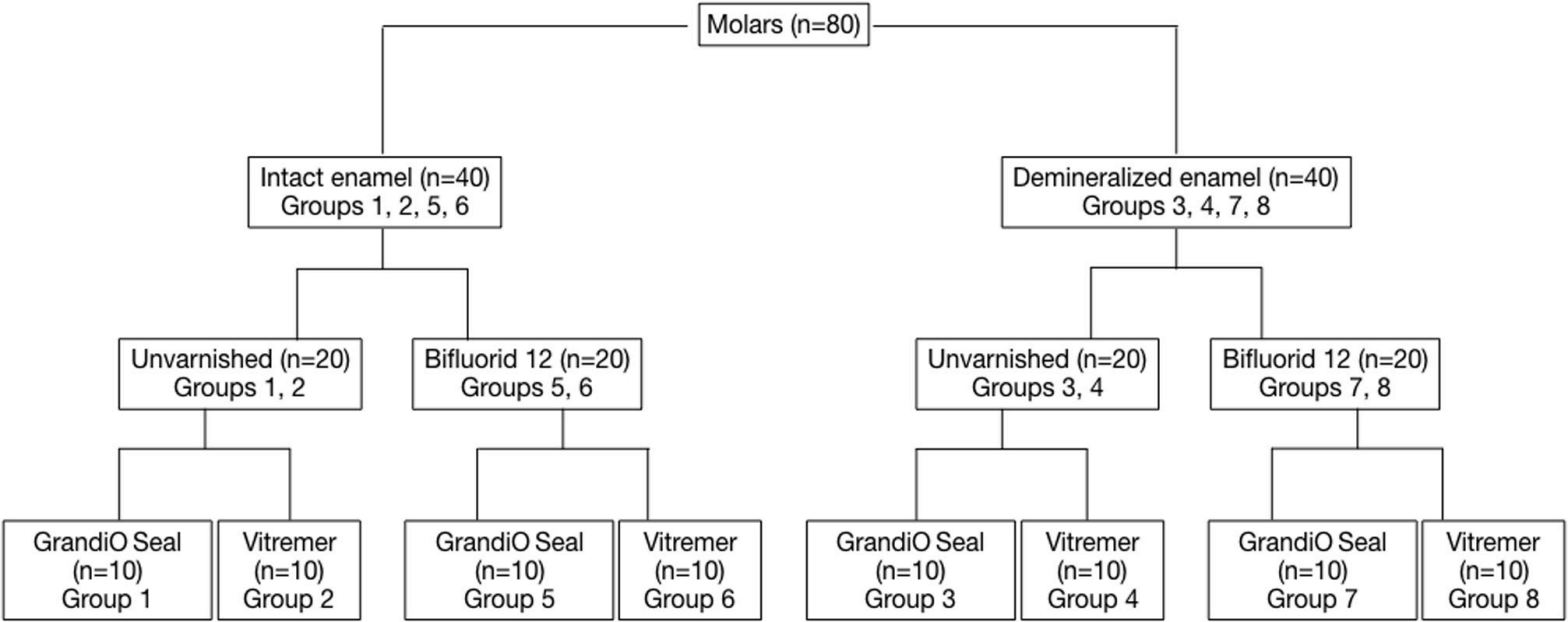
Flowchart of experimental groups.

The 80 molars were divided, using a table of random numbers, into 8 groups of 10 teeth each (Fig 1).

Groups 3, 4, 7 and 8 were placed in a demineralizing solution for 48 hours (2.2 mM calcium chloride [CaCl_2_.2H_2_O], 2.2 mM monosodium phosphate [NaH_2_PO_4_.7H_2_O], 0.05 mM lactic acid, pH adjusted to 4.5 with 50% sodium hydroxide [NaOH]) [22]. After 48 hours teeth were washed with a water spray and placed in a digital ultrasonic cleaner (R-100135, Mestra, Bilbao, Spain) with distilled water at room temperature for 1 hour.

Bifluorid 12 (VocoGmbH, Cuxhaven, Germany) was applied in groups 5, 6, 7 and 8; the product composition is detailed in Table 1, together with the other products used in the experiments. Following the manufacturer’s instructions, the Bifluorid 12 bottle was shaken before each use to mix the particles thoroughly. The varnish was applied to the enamel with a disposable applicator, left to penetrate the surface for 20 seconds, and then dried with compressed air. The teeth were then placed in artificial saliva for 24 hours and kept at a temperature of 37°C, in an incubator (JP Selecta SA, Barcelona, Spain). The composition of the saliva used as storage medium was: 1% carmellose sodium, 13% sorbitol, 0.12% potassium chloride, 0.084% sodium chloride, 0.005% magnesium chloride hexahydrate, 0.015% anhydrous calcium chloride, 0.017% dibasic potassium phosphate, and 0.1% Nigapin sodium. The saliva pH was adjusted and maintained at 6.57 [23].

**Table 1:** Product composition according to material safety data sheets (MSDS).

Product	Composition		(%)
Bifluorid 12	Ethyl acetate		50–100
	Cellulose nitrate with alcohol		10–25
	Isopentyl propionate		10–25
	Sodium fluoride		6
	Calcium fluoride		6
	Pyroxylin		<1
	Fumed silica		<1
	Eugenol		<1
	Clove oil		<1
Dentaflux Acid	Orthophosphoric acid		37
	Excipients		Till 100
Vitremer	Primer	Ethanol	44–48
		2-hydroxyethyl methacrylate (HEMA)	37–41
		Polycarboxylic acid copolymer	11–15
	Powder	Methacryloxypropyltrimethoxysilane	2–4
		Silicate and aluminosilicate mixture	95–98
	Liquid	Polycarboxylic acid copolymer	50–55
		Water	27–30
GrandiO Seal	Bis-GMA		2.5–5
	Triethylene glycol dimethacrylate		10–25
	Fumed silica		5–10

In groups 1, 3, 5 and 7, GrandiO Seal was applied (VocoGmbH, Cuxhaven, Germany). The whole exposed occlusal surface was etched with 37% orthophosphoric acid (DentaFlux, Madrid, Spain) for 20 seconds, washed with abundant water in a spray for 20 seconds and dried with dry compressed air. The sealant was applied with the applicator tip and polymerized for 20 seconds with a SmartLite LED lamp at 1250 W/cm^2^ (Dentsply, York, PA, USA).

In groups 2, 4, 6 and 8, Vitremer was applied. Pits and fissures were etched with 37% orthophosphoric acid (DentaFlux, Madrid, Spain) for 20 seconds, washed with abundant water in a spray for 20 seconds and dried with dry compressed air. The surface was brushed with Vitremer primer for 30 seconds, dried for 15 seconds, and polymerized for 20 seconds with a SmartLite LED lamp at 1250 W/cm^2^ (Dentsply, York, PA, USA). The powder and liquid were mixed at a ratio of a quarter spoonful of powder with a drop of liquid to achieve a more fluid consistency. The mixture was placed in the occlusal grooves by a probe and polymerized for 40 seconds. Finally, it was brushed with Vitremer finish gloss, which was polymerized for 20 seconds.

### Microleakage testing

After application of the sealants, the teeth were placed in artificial saliva for 20 days and kept at a temperature of 37°C in an incubator (JP Selecta SA, Barcelona, Spain), after which they were subjected to 500 cycles of thermocycling (5–55°C). After thermocycling, the specimens were submerged in a 1% solution of methylene blue for 24 hours. To prevent the penetration of methylene blue through the apical foramina, the teeth were placed vertically in a container with a metal grid. The tooth roots fit into the metal grid, and the methylene blue covered only the crown of the tooth.

### Microscopic observations

After 24 hours, the teeth were washed and dried and the crowns cut into three sections in the occlusal-cervical direction with a diamond disk with abundant water cooling (918OB, Komet, GebrBrasseler GmbH & Go.KG, Germany). Each section was examined on both sides, meaning that each tooth was examined six times. The percentage of microleakage was determined using a Sony DXC 151AP video camera connected to an Olympus SZ11 microscope using the image analysis program MIP 4 (Microm Image Processing Software, Digital Image Systems, Barcelona, Spain) at a magnification of x10. The total percentage of microleakage for each section observed was calculated using the formula: [(length of stained interface/total perimeter of the interface) x 100]. The mean of the six measurements per tooth provided the microleakage of each tooth. The mean of the 10 teeth provided the microleakage of the group.

Two blinded observers made the observations. To determine whether there were differences, the paired *t*-test was used to compare values between observers. As there were no significant differences, the mean of the values was taken as the definitive value of the group.

### Statistical analysis

The statistical analysis was performed using the SigmaStat 3.5 statistical software package (Systat Software Inc., Point Richmond, CA, USA). Microleakage values did not have a normal distribution (Kolmogorov-Smirnov test, p <0.05) or homogeneity of variance (Levene’s test, p <0.05). The Kruskal-Wallis test was used to compare differences in microleakage in the different groups. As there was a significant difference (p = <0.001), all pairwise multiple comparisons were determined using Dunn’s method.

We used a three-way ANOVA to determine the interaction between the three study factors: varnish (varnished or unvarnished enamel), type of enamel (intact or demineralized), and material (resin or ionomer). After determining the interactions, all pairwise multiple comparisons were made using the Tukey test. Statistical significance was set at p <0.01.

## Results

**Fig 2:**
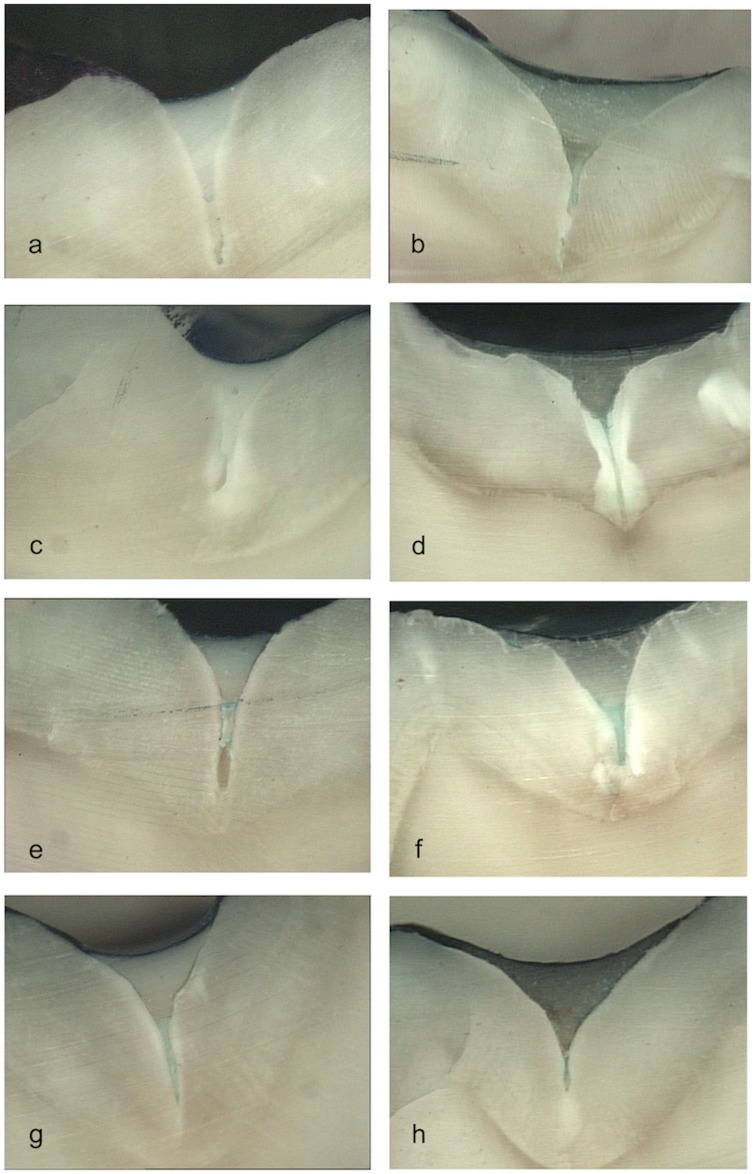
Images obtained in the stereomicroscope (x10). (a) Group 1: intact enamel + GrandiO Seal. (b) Group 2: intact enamel + Vitremer. (c) Group 3: demineralized enamel + GrandiO Seal. (d) Group 4: demineralized enamel + Vitremer. (e) Group 5: intact enamel + Bifluorid 12 + GrandiO Seal. (f) Group 6: intact enamel + Bifluorid 12 + Vitremer. (g) Group 7: demineralized enamel + Bifluorid 12 + GrandiO Seal. (h) Group 8: demineralized enamel + Bifluorid 12 + Vitremer.

There was a significant interaction between the presence or absence of Bifluorid12 and the degree of enamel mineralization (p = <0.001), i.e., the amount of microleakage in the mineralized and demineralized enamel depended on whether the enamel was varnished or not. Thus, when the enamel was not varnished, there was no difference in microleakage between intact or demineralized enamel (p = 0.055), regardless of the type of material used (GrandiO Seal or Vitremer). The least microleakage was in the unvarnished groups (groups 1–4). However, when the enamel was varnished (groups 5–8) there were differences between the intact and demineralized enamel (p <0.001), as microleakage was higher in the demineralized enamel than in the intact enamel for both LCRBS (group 7: 28.66 ± 3.44 vs. group 5: 13.32 ± 5.53) and the RMGIS (group 8: 21.14 ± 4.06 vs. group 6: 4.30 ± 1.01) (Table 2; Fig 2).

**Table 2:** Mean percentage of microleakage.

Group	Bifluorid12	Enamel	Sealant	± SD (%)	
1	-	Intact	GrandiO Seal	3.20 ± 1.34	a
2	-	Intact	Vitremer	3.90 ± 1.23	a
3	-	Demineralized	GrandiO Seal	3.29 ± 2.02	a
4	-	Demineralized	Vitremer	2.92 ± 1.16	a
5	Yes	Intact	GrandiO Seal	13.32 ± 3.53	b
6	Yes	Intact	Vitremer	4.30 ± 1.01	a
7	Yes	Demineralized	GrandiO Seal	28.66 ± 3.44	c
8	Yes	Demineralized	Vitremer	21.14 ± 4.06	c

Identical lower case letters indicate no significant differences, and different lower case letters show significant differences (P<0.01). Kruskal-Wallis test with the Dunn’s method.

There was no significant interaction but a trend to interaction between the presence or absence of varnish and the type of sealant used (GrandiO Seal or Vitremer) (p = 0.027) was observed. The behaviour of the two materials differed according to whether the enamel was varnished or not. In the unvarnished enamels, both materials showed a similar performance, but in the varnished enamels GrandiO Seal (groups 5 and 7) always showed a greater degree of microleakage than Vitremer (groups 6 and 8) (Table 2; Fig 2).

However, there was no significant interaction between the degree of enamel mineralization and the type of sealant used (p = 0.955). That is, the degree of mineralization did not differentiate between the performance of the two types of sealants (Table 2; Fig 2).

## Discussion

The aim of this study was to assess the influence of the application of a high fluoride varnish (Bifluorid 12, Voco) on microleakage of a LCRBS (GrandiO Seal, Voco) and a RMGIS (Vitremer, 3M) placed on demineralized enamel.

At present, there is no standardized method for the *in vitro* evaluation of microleakage of pit and fissure sealants, making it difficult to compare results between studies [24] We expressed microleakage as the percentage of penetration of methylene blue along the enamel-sealant interface of pits and fissures, as we believe this is more accurate than the use of dichotomous or numerical scales. Numerical scales are not standardized and each has different degrees to express the depth reached by the dye; and dichotomous scales only evaluate the presence or absence of marginal adaptation [25,26]

No sealant remains perfectly adapted to the dental structure over time, and all will suffer some degree of microleakage. This is because the coefficient of thermal expansion of sealants is 2–4 times greater than that of enamel. Therefore, the constant temperature changes in the oral cavity give rise to the formation of gaps that facilitate the penetration of bacteria at the interface between the sealant and the enamel [16].

Our results show that the microleakage of LCRBS and RMGIS was similar when placed on the enamel without a fluoride varnish covering. The same results were observed by Markovic et al. [27] using a fluorine-releasing resin sealant (Helioseal F) and a glass ionomer modified with acidic monomers (Fuji Triage), and Pardi et al. [28] using a self-curing unfilled LCRBS (Delton), a fluid composite (Filtek Flow), a fluid compomer (Dyract Flow) and a RMGIS (Vitremer), as they detected no significant differences in microleakage between the different materials.

In contrast, Ganesh and Shobha [29], Gunjal, Nagesh and Raju [30], Rirattanapong, Vongsavan, and Surarit [26] observed greater microleakage in teeth sealed with a high-density glass ionomer (Fuji VII) when compared with resin sealants, probably due to a lower penetration depth of the high–density ionomers in pits and fissures. The lower microleakage of resin sealants compared with glass ionomer sealants could be due to the lack of application of an acid etch prior tosealant placement. We did use etching with orthophosphoric acid for 20 seconds prior to the placement of the glass ionomer primer, and this allowed the microleakage of Vitremer in unvarnished enamels to be similar to that of GrandiO Seal. According to Fracasso et al. [31], acid etching of the enamel causes increased penetration of RMGIS (Vitremer), even in the less diluted concentration (1:1, instead of 1:¼), since the micromechanical adhesion-retention of acid etching is better than the chemical adhesion of RMGIS.

If an adhesive is added to acid etching, microleakage decreases and retention increases. Cheque Bernardo et al. [32] placed Vitremer after etching with 35% orthophosphoric acid for 30 seconds and applying Scotchbond Multipurpose adhesive, and obtained a significantly higher retention rate than when RMGIS was applied in a conventional manner. The retention rate was similar to that of resin sealers based on Bis-GMA (88% at 12 months).

Our results showed that the use of fluoride varnish resulted in differences in the performance of the two types of sealants. Microleakage in the groups sealed with GrandiO Seal was greater than in those sealed with Vitremer. Contraction of the polymerization of LCRBS may lead to the formation of gaps between the sealant and enamel when there is not good penetration of the sealant. The varnish, in our case, would be creating an obstacle to the penetration of the resin sealant in the micropores created by acid etching, worsening marginal sealing and favouring microleakage [33]. The difference in performance with RMGIS was probably due to the fact that Vitremer only contains a small percentage of resin, resulting in less polymerization contraction, which is due to the setting of the acid-base reaction, which would be compensated for by the absorption of water from the buccal medium typical of glass ionomers [18].

The presence of fluoride varnish also determined greater microleakage when the sealants were applied in the demineralized enamel compared with the intact enamel, regardless of the type of sealant used. However, Kantovitz et al. [34] did not observe this difference between intact enamel and demineralized enamel, when they studied the marginal adaptation of a fluorine resin sealant with another without fluorine, using a 5% NaF varnish (Duraphat).

Although this type of *in vitro* study has the advantage of being able to control determining factors such as the degree of demineralization, the conditions of application of the materials and the temperature, *in vivo* studies would be necessary to determine the marginal adaptation and retention of pit and fissure sealants in varnished and unvarnished demineralized enamel.

Within the limitations of this in vitro study, it may be affirmed that the use of a fluoride varnish (Bifluorid 12) increased microleakage of pit and fissure sealants when applied at 24 hours. In demineralized enamel it is preferable to use the sealant directly, since application of the fluoride varnish increases microleakage, and the increase is greater for the LCRBS (GrandiO Seal) than for the RMGIS (Vitremer).
